# Facilitation of glutamate, but not GABA, release in Familial Alzheimer's *APP* mutant Knock‐in rats with increased β‐cleavage of APP

**DOI:** 10.1111/acel.13033

**Published:** 2019-09-09

**Authors:** Marc D. Tambini, Wen Yao, Luciano D'Adamio

**Affiliations:** ^1^ Department of Pharmacology, Physiology & Neuroscience New Jersey Medical School Brain Health Institute Jacqueline Krieger Klein Center in Alzheimer's Disease and Neurodegeneration Research Rutgers, The State University of New Jersey Newark NJ USA

**Keywords:** Alzheimer's Disease, APP, BACE1, glutamate release, synaptic transmission

## Abstract

Amyloid precursor protein (APP) modulates glutamate release via cytoplasmic and intravesicular interactions with the synaptic vesicle release machinery. The intravesicular domain, called ISVAID, contains the BACE1 cleavage site of APP. We have tested the functional significance of BACE1 processing of APP using *App*‐Swedish (*App* ^*s*^) knock‐in rats, which carry an *App* mutation that causes familial Alzheimer's disease (FAD) in humans. We show that in *App* ^*s*^ rats, β‐cleavage of APP is favored over α‐cleavage. *App* ^*s*^ rats show facilitated glutamate, but not GABA, release. Our data support the notion that APP tunes glutamate release, and that BACE1 cleavage of the ISVAID segment of APP facilitates this function. We define this phenomenon as BACE1 on APP‐dependent glutamate release (BAD‐Glu). Unsurprisingly, *App* ^*s*^ rats show no evidence of AD‐related pathology at 15 days and 3 months of age, indicating that alterations in BAD‐Glu are not caused by pathological lesions. The evidence that a pathogenic APP mutation causes an early enhancement of BAD‐Glu suggests that alterations of BACE1 processing of APP in glutamatergic synaptic vesicles could contribute to dementia.

## 1 INTRODUCTION

Amyloid precursor protein (APP) is a ubiquitously expressed type I membrane protein. In the central nervous system, APP is mainly expressed in neurons (Guo et al., 2012). The following evidence supports the hypothesis that APP participates in the modulation of glutamatergic synaptic vesicles (SV) release: (a) APP is found in SV (Del Prete, Lombino, Liu & D'Adamio, 2014; Groemer et al., 2011; Yao, Tambini, Liu & D'Adamio, 2019); (b) endogenous APP is present in fractions highly enriched in presynaptic termini (Groemer et al., 2011; Lundgren et al., 2015; Yao et al., 2019); (c) the in vivo interactome of APP in brain reveals a protein network with SV proteins (Kohli et al., 2012; Norstrom, Zhang, Tanzi & Sisodia, 2010); (d) APP possess two domains, one cytosolic and one intravesicular, that bind SV proteins (Del Prete et al., 2014; Fanutza, Del Prete, Ford, Castillo & D'Adamio, 2015; Yao et al., 2019); (e) interfering with these interactions modulates glutamatergic SV release (Fanutza et al., 2015; Yao et al., 2019).

The intravesicular SV‐binding domain of APP, called ISVAID (Yao et al., 2019), contains the β‐ and α‐secretase cleavage sites of APP, and this suggests the possibility of α/β‐secretase‐mediated modulation of SV function. Indeed, interactome and electrophysiology evidence suggests that cleavage of APP in the ISVAID segment by either β‐ or α‐secretase may reduce or abolish the intravesicular interactions of APP with SV proteins, leading to facilitation of glutamatergic SV release (Yao et al., 2019). The acidic pH of SVs favors β‐cleavage, and there is an enrichment of β‐cleaved APP metabolites in SV subcellular fractionations (Del Prete et al., 2014). Thus, it is reasonable to hypothesize that processing of APP by β‐secretase, rather than α‐secretase, may cut inside the ISVAID of APP and destabilize these interactions, thereby facilitating excitatory neurotransmission. The evidence that a BACE1 inhibitor causes strong reduction in the frequency of sEPSC/mEPSC (Filser et al., 2014) and that BACE1 KO mice show an increase in PPF ratio, which is indicative of a reduction in presynaptic release (Wang, Song, Laird, Wong & Lee, 2008), are consistent with this hypothesis.

However, β‐secretase may process several synaptic substrates that may affect synaptic transmission; thus, whether the effects of pharmacological and genetic ablation of β‐secretase activity are due to inefficient β‐processing of APP is unclear. To directly test this hypothesis, a system in which App processing by BACE1 is increased without changing BACE1 activity is needed. A naturally occurring *APP* mutant allele, the pathogenic Swedish *APP* allele, codes for an APP protein (herein called APPSw) carrying the amino acids substitutions K670N/M671L. These mutations, which are localized at the NH_2_‐terminus of the β‐cleavage site of APP, cause increased cleavage of APP by β‐secretase (Citron et al., 1992, 1994; Johnston et al., 1994). Thus, a model organism in which APPSw replaces wild‐type APP should be apt to test whether APP processing by β‐secretase facilitates excitatory neurotransmission.

To test this hypothesis, we introduced the Swedish *APP* mutations into the genomic *App* rat locus to generate *App* ^*S*^ Knock‐in (KI) rats. Rat and human APP differ in the Aβ region by 3 amino acids. Given that aggregated or oligomeric forms of Aβ are by and large considered the main pathogenic entity in AD and given that human Aβ may have higher propensity to form toxic Aβ species as compared to rodent Aβ, together with the Swedish mutations we introduced mutations to “humanize” the rat Aβ sequence. As controls, we produced rats carrying only the humanized Aβ sequence (*App* ^*h*^ rats, APPh protein). We choose a KI approach because (a) KIs mimic the genetics of familial dementia and make no assumption about pathogenic mechanisms (except the unbiased genetic one); (b) expression of mutant genes is controlled by endogenous regulatory elements in physiological quantitative‐spatial‐temporal manner, thereby allowing us to test the hypothesis in a biologically relevant model organism system.

## 2 RESULTS

### 2.1 *App* mRNA expression is normal in *App* ^*h*^ and *App* ^*s*^ KI rats but is greatly reduced in *App* ^δ*7*^ rats

**Figure 1:**
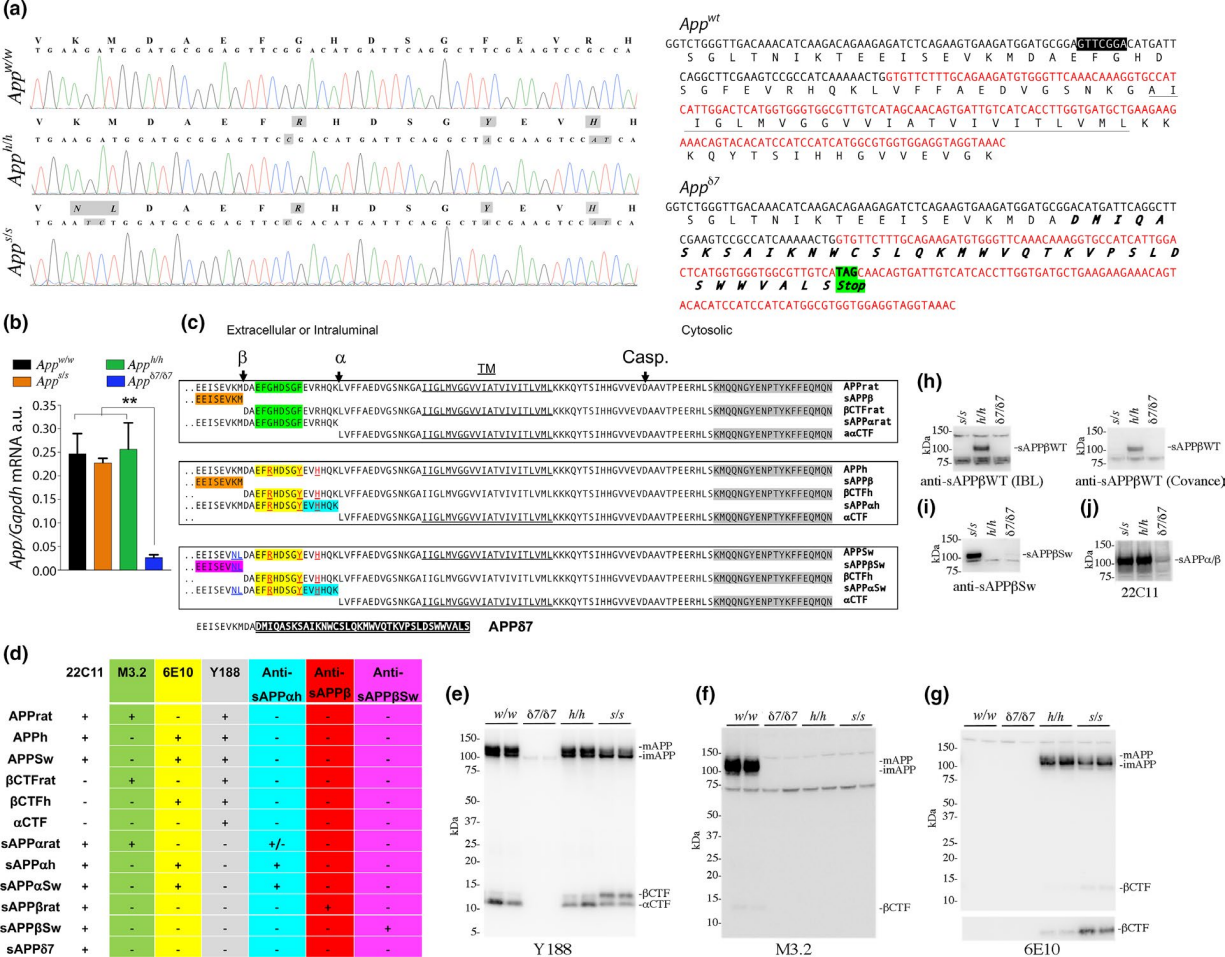
Characterization of *App* ^*h*^, *App* ^*s*^, and *App* ^δ*7*^ KI rats. (a) Left panel. To verify that the humanizing and Swedish mutations were correctly inserted in the *App* exon‐16, we amplified by PCR the *App* gene exon‐16 from *App* ^*w/w*^ *, App* ^*h/h*^, and *App* ^*s/s*^ rats. Sequencing of the PCR products shows that the humanizing mutations and the humanizing plus Swedish mutations were correctly inserted into the *App* ^*h/h*^ and *App* ^*s/s*^ genomes, respectively. Sequencing analysis of genomic DNA confirms the expected G to C, T to A, and GC to AT substitution in *App* ^*h/h*^ rats and the GA to TC, G to C, T to A, and GC to AT substitution in *App* ^*s/s*^ rats. Substituted nucleotides are highlighted in gray. The amino acid sequences are indicated above the DNA sequences and the amino acid substitutions introduced by the mutations are highlighted in gray (GA to TC=KM to NL; G to C=G to R; T to A=Y to F; and GC to AT=R to H). Right panel. Predicted sequences of WT and *App* ^δ7^ cDNAs and proteins. For space reasons, only Exon 16 (black) and Exon 17 (red) are shown. The 7‐bp deletion (in white characters and boxed in black), the transmembrane domain of APP (underlined), and the novel predicted COOH‐terminal of sAPP ^δ7^ (*bold and italic*) are indicated. (b) Levels of *App* mRNA were measured in 21‐day‐old *App* ^*w/w*^, *App* ^δ*7/*δ*7*^ *, App* ^*h/h*^, and *App* ^*s/s*^ rats (2 females and 3 males for each genotype). *App* mRNA expression was normalized to *Gapdh* mRNA expression. The δ*7* mutant allele was a product of aberrant homology‐directed repair but is useful because this mutation will either produce a truncated soluble APPδ7 protein (sAPPδ7) or a hypomorphic allele. Data were analyzed by ordinary one‐way ANOVA followed by post hoc Tukey's multiple comparisons test when ANOVA showed statistically significant differences and presented as average (*App*/*Gapdh*) ± *SEM*. (c) Schematic representation of APPWT rat, APPh, APPSw, and the metabolites derived from α‐ and β‐secretase processing. The amino acid changes that humanize the Aβ region of are in red and underlined (G>R, F>Y, R>H); the amino acids changes that introduce the Swedish mutation are in blue and underlined (K>N, M>L); the epitopes recognized by antibodies are highlighted as follows: Y188 is highlighted in gray, M3.2 is highlighted in green, 6E10 highlighted in yellow, anti‐sAPPα is highlighted in cyan, anti‐sAPPβWT is highlighted in red, and anti‐sAPPβSw is highlighted in magenta. The αCTF and βCTF derived from APPh and APPSw are identical; thus, in the paper, we will refer to both as αCTF or βCTF. The *App* ^δ*7*^ allele could produce sAPPδ7: The sequence in white highlighted in black indicates the novel amino acid sequence produced by the deletion of 7 bp, which causes a frameshift. Casp. Indicates the site of cleavage of APP in the cytoplasmic region by caspases, which leads to the generation of JCasp (Gervais et al., 1999; Pellegrini, Passer, Tabaton, Ganjei & D'Adamio, 1999). (d) Summary of the expected immunoreactivities of the anti‐APP antibodies used in this study: + = positive reactivity; − = no reactivity. (e) Western blot analysis of postnuclear supernatant isolated from *App* ^*w/w*^, *App* ^δ*7/*δ*7*^, *App* ^*h/h*^, and *App* ^*s/s*^ rats with Y188 (detects mAPP, imAPP, αCTF, and βCTF from all animals except the *App* ^δ*7/*δ*7*^ rats). (f) M3.2 (detects only rat WT mAPP, imAPP, and βCTF). (g) 6E10 (detects only mAPP, imAPP, and βCTF carrying the humanizing mutation). (h) Western blot analysis of brain soluble fractions with two anti‐sAPPβWT antibodies (which detect sAPPβ *App* ^*h/h*^ rats only). (i) An anti‐sAPPβSw antibody (detects sAPPβ in *App* ^*s/s*^ rats only). (j) 22C11 (detects sAPPβ and sAPPα in *App* ^*h/h*^ and *App* ^*s/s*^ rats). (k) Anti‐sAPPα (detects sAPPα in *App* ^*h/h*^ and *App* ^*s/s*^ rats)

Founder (F0) rats carrying the *h, s,* and δ*7* mutations were generated as described in the Experimental Procedures section. F0‐*App* ^*h/*δ*7*^ and F0‐*App* ^*s*^ rats were crossed to WT (*App* ^*w/w*^) Long Evans rats to generate F1‐*App* ^δ*7/w*^, F1‐*App* ^*h/w*^, and F1‐*App* ^*s/w*^ rats. The δ*7* mutant allele was a product of aberrant homology‐directed repair but is useful. As shown in Figure 1a, this 7‐bp deletion in exon 16 causes a frameshift that would produce a truncated soluble protein (sAPPδ7, missing the transmembrane region of APP) with a novel COOH‐terminal sequence. Alternatively, this mutation could also produce a hypomorphic allele. F1‐*App* ^δ*7/w*^, F1‐*App* ^*h/w*^, and F1‐*App* ^*s/w*^ rats were crossed to WT Long Evans to generate F2‐*App* ^δ*7/w*^, F2‐*App* ^*h/w*^, and F2‐*App* ^*s/w*^ rats. These crossing were repeated three more times to obtain F5‐*App* ^δ*7/w*^, F5‐*App* ^*h/w*^, and F5‐*App* ^*s/w*^ rats. The probability that F5 rats carry unidentified off‐target mutations (except those, if present, on Chr. 11) is ~1.5625%. Male and female F5‐*App* ^δ*7/w*^, F5‐*App* ^*h/w*^, and F5‐*App* ^*s/w*^ rats were crossed to obtain *App* ^δ*7/*δ*7*^ *, App* ^*h/h*^, and *App* ^*s/s*^ rats.

To verify that the humanizing and Swedish mutations were correctly inserted into *App* exon‐16, we amplified by PCR the *App* gene exon‐16 from *App* ^*w/w*^ *, App* ^*h/h*^, and *App* ^*s/s*^ rats. Sequencing of the PCR products shows that the humanizing mutations and the humanizing plus Swedish mutations were correctly inserted in the *App* ^*h/h*^ and *App* ^*s/s*^ genomes, respectively (Figure 1a).

To determine whether the introduced mutations disrupt *App* expression, we examined *App* mRNA levels in 21‐day‐old *App* ^*w/w*^, *App* ^δ*7/*δ*7*^ *, App* ^*h/h*^, and *App* ^*s/s*^ rats (2 females and 3 males for each genotype). In *App* ^δ*7/*δ*7*^ rats, *App* mRNA was virtually absent, indicating that the *App* ^δ*7*^ mutation is a hypomorphic mutation. In contrast, expression of *App* in *App* ^*h/h*^ and *App* ^*s/s*^ brains was comparable to that detected in *App* ^*w/w*^ rats (Figure 1b). Following are the statistical data obtained by ordinary one‐way: ANOVA summary: *F* = 9.588, *p *=* *.0007 (significant = ***). Post hoc Tukey's multiple comparisons test: *App* ^*w/w*^ vs *App* ^*s/s*^, *p* = .9800 (not significant); *App* ^*w/w*^ vs *App* ^δ*7/*δ*7*^, *p* = .0022 (significant=**); *App* ^*w/w*^ vs *App* ^*h/h*^, *p* = .9970 (not significant); *App* ^*s/s*^ vs *App* ^δ*7/*δ*7*^, *p* = .0048 (significant = **); *App* ^*s/s*^ vs *App* ^*h/h*^, *p* = .9352 (not significant); *App* ^*h/h*^ vs *App* ^δ*7/*δ*7*^, *p* = .0015 (significant = **).

### 2.2 The proteins encoded by the *App* ^*h*^ and *App* ^*s*^ alleles contain the humanizing and Swedish mutations, while the *App* ^δ*7*^ is an hypomorphic *App* allele

To verify whether the protein products of the *App* ^*h*^ and *App* ^*s*^ alleles contain the humanizing mutations, we used the following anti‐APP antibodies. Y188, a rabbit polyclonal raised against the COOH‐terminal 20 amino acids of APP, an epitope that is unchanged by the humanizing and Swedish mutations. M3.2, a mouse monoclonal raised against the rat APP sequence between the β‐ and α‐secretase cleavage sites (DAEFGHDSGFEVRHQK); this antibody will only recognize APP molecules containing the rat Aβ sequence. 6E10, a mouse monoclonal raised against the corresponding domain of human APP (DAEFRHDSGYEVHHQK, the 3 amino acid differences with the rat sequence are underlined); this antibody will only recognize APP molecules containing the human Aβ sequence. The specificities of these antibodies are summarized in Figure 1c,d. Y188 detects mAPP, imAPP, βCTF, and αCTF in *App* ^*w/w*^ *, App* ^*h/h*^, and *App* ^*s/s*^ brains (Figure 1e). M3.2 detected mAPP, imAPP, and βCTF in *App* ^*w/w*^ brains (Figure 1f). Conversely, 6E10 detected mAPP, imAPP, and βCTF only in *App* ^*h/h*^ and *App* ^*s/s*^ rats (Figure 1f). As expected, none of these antibodies gave any specific signal in *App* ^δ*7/*δ*7*^ samples. Thus, APPh and APPSw, the protein products of the *App* ^*h*^ and *App* ^*s*^ alleles, contain the humanized Aβ sequence.

To verify that APPSw contained the Swedish mutations, we used two antibodies raised against the COOH terminus of wild‐type human sAPPβ (sAPPβWT), one from IBL the other from Covance, and an antibody raised against the COOH terminus of Swedish sAPPβ (sAPPβSw) (the KM>NL mutations are in the 2 COOH‐terminal residues of sAPPβ). The two anti‐sAPPβWT should detect sAPPβ produced from APPh (called sAPPβh) but not sAPPβ produced from APPSw (called sAPPβSw); conversely, the anti‐sAPPβSw should only detect sAPPβSw and not sAPPβh. The specificities of these antibodies are summarized in Figure 1c,d. As expected, analysis of soluble brain fractions (S70) isolated from one *App* ^*h/h*^, one *App* ^*s/s*^, and one *App* ^δ*7/*δ*7*^ showed that the two anti‐sAPPβWT detected sAPPβh only (Figure 1h), while the anti‐sAPPβSw only detected sAPPβSw (Figure 1i); none of the antibodies gave a positive signal in the *App* ^δ*7/*δ*7*^ sample.

To complete our preliminary biochemical analysis of soluble fractions, we performed Western blots with 22C11, a monoclonal antibody raised against the ectodomain of APP that will recognize all sAPP and sAPPβ species as well as sAPPδ7, and an anti‐sAPPα monoclonal antibody, which is raised against the COOH terminus of sAPPα and is specific for sAPPα (see Figure 1c,d). 22C11 (Figure 1j) detected equal amounts of sAPP species in both *App* ^*h/h*^ and *App* ^*s/s*^ but gave no specific signal in the *App* ^δ*7/*δ*7*^ sample (no sAPPδ7 detected), confirming that δ*7* is a hypomorphic *App* allele. Anti‐sAPPα detected both sAPPαh (sAPPα derived from APPh) and sAPPαSw (sAPPα derived from APPSw) (Figure 1k). Overall, these data indicate that (a) the pattern of immunoreactivity of APP and its metabolites confirm that the predicted mutations have been properly integrated into APPh and APPSw proteins; (b) *App* ^δ*7*^ is a hypomorphic *App* allele.

### 2.3 Increased β‐processing of APP in Familial Alzheimer disease *App*‐Swedish Knock‐in rats

**Figure 2:**
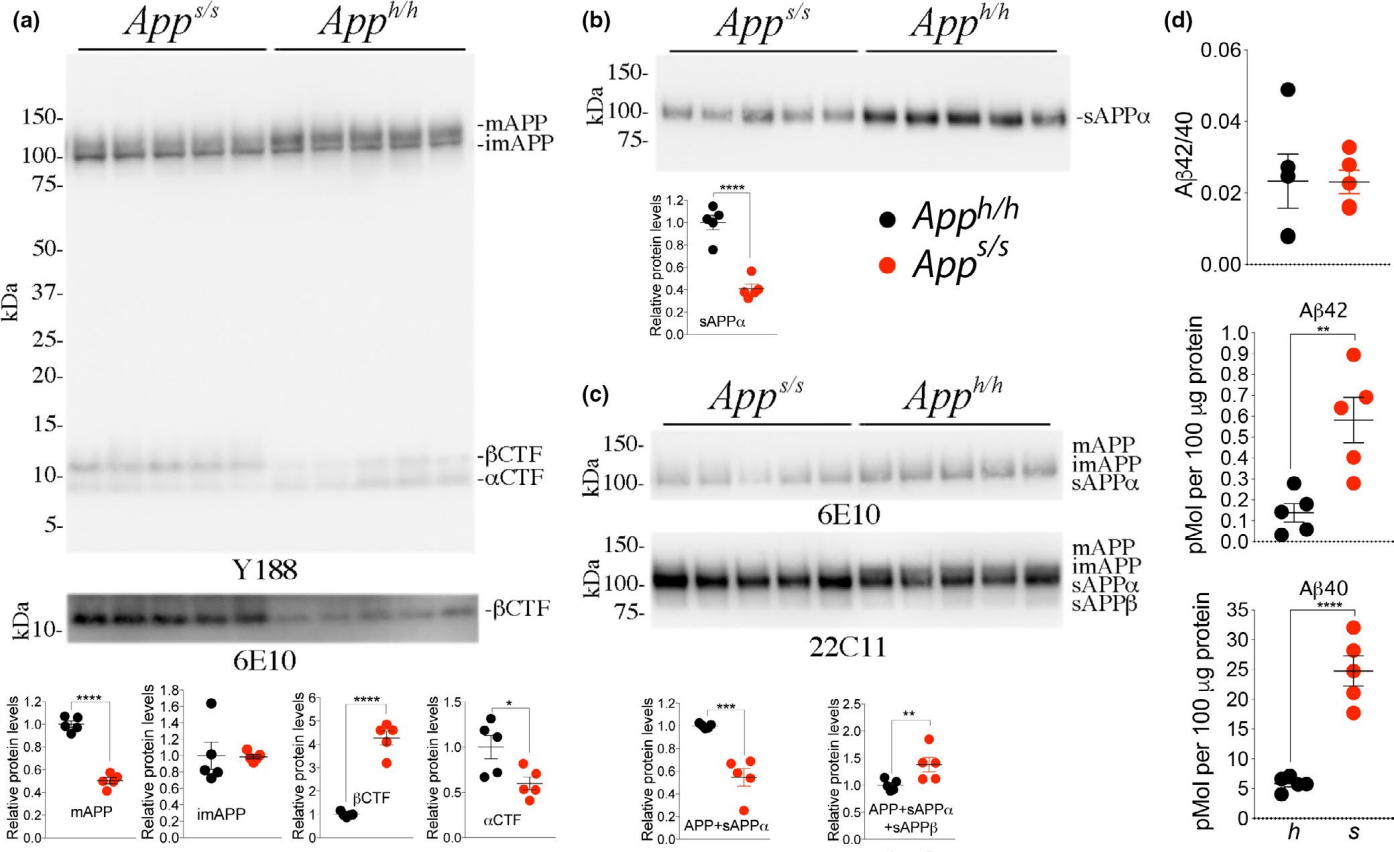
Increased processing by β‐secretase and decreased processing by α‐secretase of APPSw. To test whether the *App* ^*s/s*^ rats present the expected changes in APP metabolism, we analyzed brain samples isolated from 21‐day‐old *App* ^*h/h*^ and *App* ^*s/s*^ rats, 2 females and 3 males for each genotype. (a) Western blot (WB) of postnuclear supernatant from *App* ^*s/s*^ and *App* ^*h/h*^ rats with Y188 and 6E10 (bottom). (b) WB analysis of soluble brain fractions with an ant‐sAPPα antibody showed that levels of sAPPα are significantly lower in *App* ^*s/s*^ compared to *App* ^*h/h*^ brains. (c) Because sAPPβh and sAPPβSw cannot be detected by the same antibody (see Figures 3a,b and 4d,e), to compare sAPPβh and sAPPβSw amounts, we used 6E10 and 22C11. Signal intensity was quantified with Image Lab software (Bio‐Rad). Quantification is shown on the right. (d) Aβ is produced by γ‐cleavage of βCTF, which is increased in *App* ^*s/s*^ brains. ELISA measurements of endogenous Aβ40 and Aβ42 in brain homogenates of 21‐day‐old animals. Levels of steady‐state endogenous Aβ40 and Aβ42 are increased in *App* ^*s/s*^ brains as compared to *App* ^*h/h*^ brains. The Aβ42/Aβ40 ratio did not change. These ELISA kits are specific for human Aβ40‐42, as attested by the fact that they gave no signal on brain homogenates from *App* ^*w/w*^ (expressing rat Aβ) and *App* ^δ*7/*δ*7*^ (expressing no Aβ) rats (not shown). Data are represented as mean ± *SEM*. Data were analyzed by Student's t test and shown as average ± *SEM*. **p <* *.01; ****p <* *.0001

As noted, several reports indicate that APPSw is a better β‐secretase substrate as compared to WT APP (Citron et al., 1992, 1994; Johnston et al., 1994). To test whether these APP metabolic changes are reproduced in our KI rats, we analyzed brain samples isolated from 21‐day‐old *App* ^*h/h*^ and *App* ^*s/s*^ rats, two females and three males for each genotype. *App* ^*s/s*^ rats showed increased βCTF and decreased αCTF/mAPP levels—while levels of imAPP were unchanged (Figure 2a). α‐Secretase cleaves mAPP in the secretory pathway and on the plasma membrane releasing sAPPα into the extracellular fluid. WB analysis of soluble brain fractions showed that levels of sAPPα are significantly lower in *App* ^*s/s*^ compared to *App* ^*h/h*^ brains (Figure 2b). β‐Secretase cleaves mAPP in acidic intracellular organelles, such as late endosomes and synaptic vesicles (SV), and a fraction of sAPPβ is released extracellularly during exocytosis. Thus, significant amounts of sAPPβ are present in both intra‐ and extracellular compartments. Hence, total brain homogenates were used to quantify sAPPβ levels. Because sAPPβh and sAPPβSw cannot be detected by the same antibody (Figures 1c, d, h and i), to compare sAPPβh and sAPPβSw amounts we used 6E10 and 22C11 antibodies: 6E10 detects imAPP, mAPP, and sAPPα proteins; 22C11 detects imAPP, mAPP, sAPPα, and sAPPβ proteins. These APP metabolites are close in size cannot be separated by SDS–PAGE. As shown in Figure 2c, the imAPP + mAPP + sAPPα signal revealed by 6E10 was significantly lower in *App* ^*s/s*^ as compared to *App* ^*h/h*^ brains. This is consistent with the finding that mAPP (Figure 2a) and sAPPα (Figure 2b) are significantly lower in *App* ^*s/s*^ brains. In contrast, the signal revealed by 22C11 (imAPP + mAPP + sAPPα + sAPPβ) was significantly higher in *App* ^*s/s*^ animal (Figure 2c). Hence, levels of sAPPβSw must be significantly higher than levels of sAPPβh. Data were analyzed by Student's t test, and significant differences are shown in the Figure (*p < .05; **p < .01, ***p < .001, ****p < .0001). In summary, the evidence that *App* ^*s/s*^ brains contain reduced amounts of αCTF/sAPPα and increased levels of βCTF/sAPPβ, indicates that APPSw is cleaved more efficiently by β‐secretase and less efficiently by α‐secretase as compared to APPh. Whether this reduction in sAPPα is due to altered affinity of APPSw for α‐secretase and/or reduced availability of substrate (APPSw) for α‐processing remains to be determined.

Aβ is produced by γ‐cleavage of βCTF, which is increased in *App* ^*s/s*^ brains, thus explaining why *App* ^*s/s*^ rats produce significantly more human Aβ40 and Aβ42 as compared to control animals (Figure 2d). The Aβ42/Aβ40 ratio did not change: This is predictable since the βCTFs derived from APPh and APPSw are identical and γ‐processing of βCTF should not change. Overall, *App* ^*s*^ KI recapitulates the biochemical changes of human APPSw metabolism, that is increased processing by β‐secretase (Citron et al., 1992, 1994; Johnston et al., 1994). Also, we describe previously unknown decreased processing of APPSw by α‐secretase and decrease in mAPPSw levels.

### 2.4 Increased glutamate, but not GABA, release in *App* ^*s*^ rats

**Figure 3:**
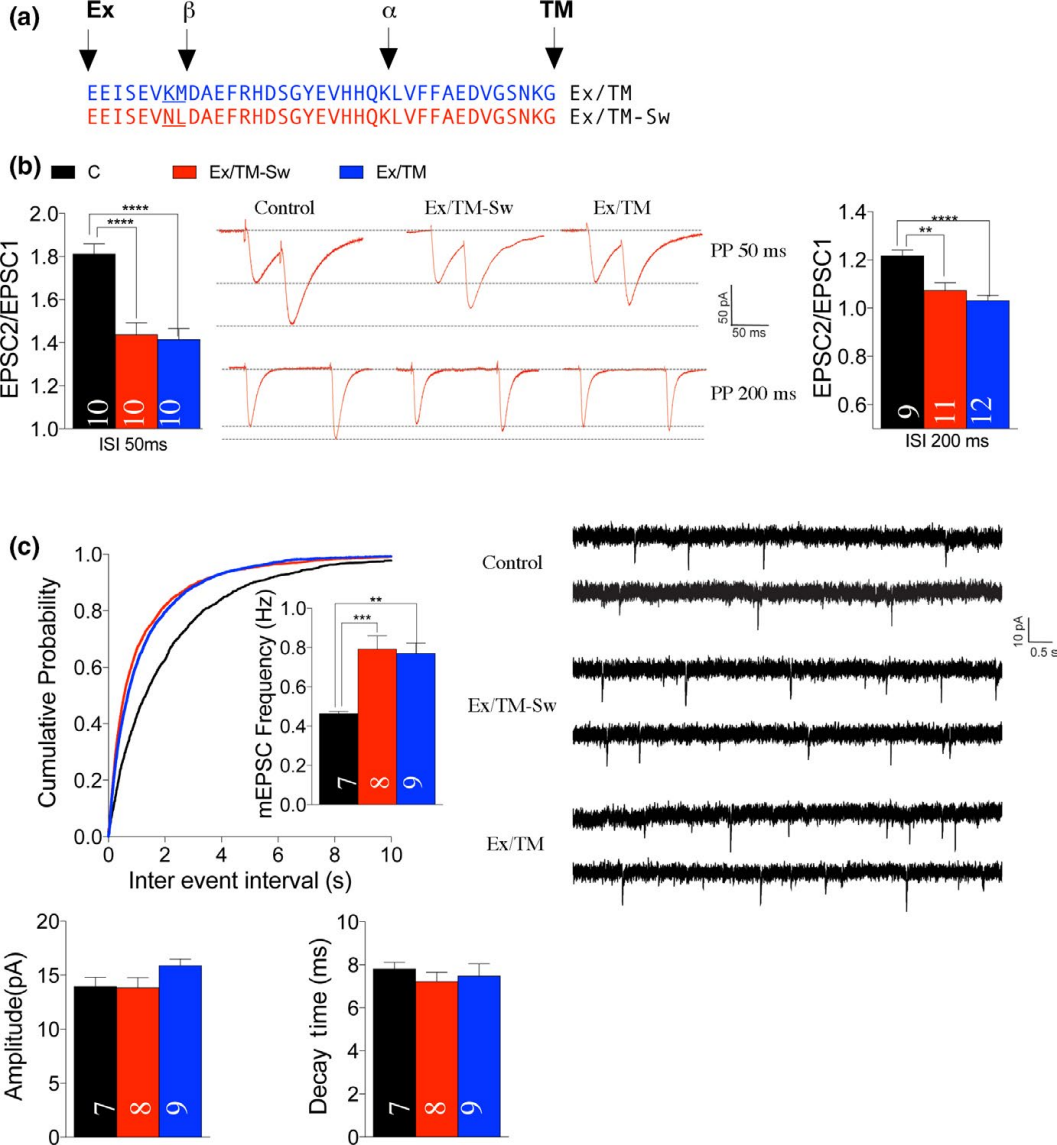
The Swedish mutations do not alter the effect of Ex/TM on glutamate release. (a) Sequence of the Ex/TM and Ex/TM‐Sw peptides. (b) Average PPF at 50 and 200 ms ISI. Representative traces of EPSCs evoked at 50 ms ISI are shown. Ex/TM and Ex/TM‐Sw significantly decrease PPF. (c) Ex/TM and Ex/TM‐Sw significantly increase mEPSC frequency. Amplitudes and decay time of mEPSCs were not changed by these peptides. Representative recording traces of mEPSCs are shown. Data were analyzed by ordinary one‐way ANOVA followed by post hoc Tukey's multiple comparisons test when ANOVA showed statistically significant differences. Four male and four female rats were used for each group. The number of recordings analyzed for each group is indicated inside the bars. All data represent means ± *SEM*

As noted above, APPSw carries the amino acids substitutions K670N/M671L. The K670 and M671 are positioned in a critical ISVAID region (Yao et al., 2019) and could modify the function of the ISVAID independently of the mutations’ effects on β‐processing. To test for this, we treated brain slices with either Ex/TM or Ex/TM‐Sw, which carries the Swedish mutations (Figure 3a), and found that both peptides reduced paired‐pulse facilitation (PPF) (Figure 3b) [ANOVA summary of PPF at 50 ms ISI: *F* = 19.91, *p* < .0001 (significant = ****); post hoc Tukey's multiple comparisons test: C vs Ex/TM‐Sw, *p* < .0001 (significant = ****); C vs Ex/TM, *p* < .0001 (significant = ****); Ex/TM vs. Ex/TM‐Sw, *p* = .9502 (not significant); ANOVA summary of PPF at 200 ms ISI: *F* = 13.48, *p* < .0001 (significant = ****); post hoc Tukey's multiple comparisons test: C vs Ex/TM‐Sw, *p* = .0018 (significant = **); C vs Ex/TM, *p* < .0001 (significant = ****); Ex/TM vs. Ex/TM‐Sw, *p* = .4580 (not significant)] and increased miniature excitatory postsynaptic currents (mEPSC) frequency [[ANOVA summary: *F* = 12.17, *p* = .0003 (significant = ***); post hoc Tukey's multiple comparisons test: C vs Ex/TM‐Sw, *p* = .0006 (significant = ***); C vs Ex/TM, *p* = .0010 (significant = **); Ex/TM vs. Ex/TM‐Sw, *p* = .9437 (not significant)], but not amplitude [ANOVA summary: *F* = 2.324, *p* = .1225 (not significant)] and decay time [ANOVA summary: *F* = 0.3776, *p* = .6900 (not significant)] (Figure 3c). These results suggest that the Swedish mutations would not alter the function of the ISVAID by a structural change in the primary amino acid sequence but by its increased affinity for Bace1 and β‐cleavage.

**Figure 4:**
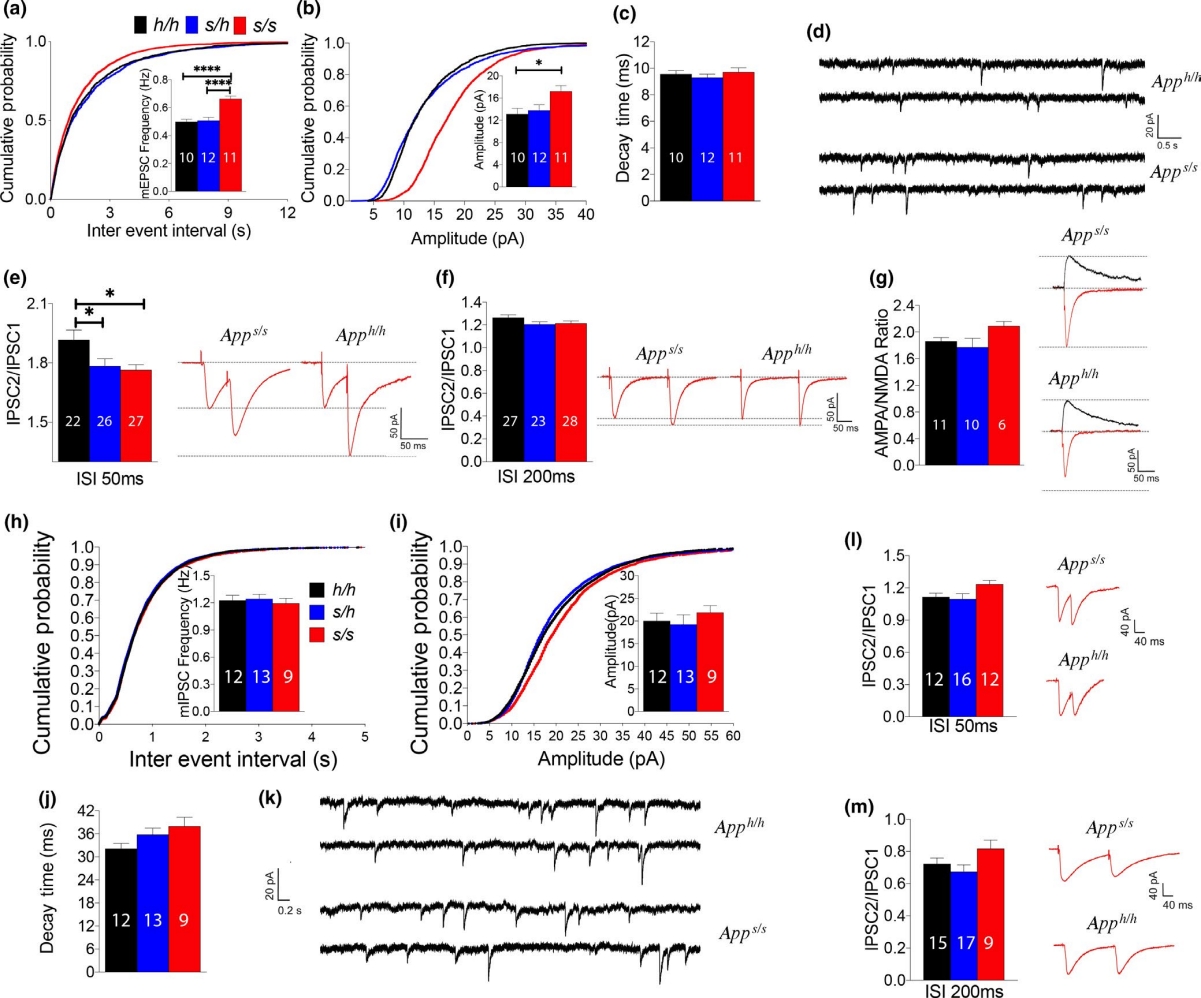
Knock‐in *App* ^*s/s*^ rats show increased glutamatergic, but not GABAergic, transmission at SC–CA3>CA1 pyramidal cell synapses. (a) *App* ^*s/s*^ rats show significantly increased frequency of mEPSC. (b) *App* ^*s/s*^ rats show significantly increased amplitude of mEPSC. (c) Decay time of mEPSCs was not changed by the Swedish mutation. (d) Representative recording traces of mEPSCs. (e) Average PPF at 50 ms ISI. Representative traces are shown on the right of the panel. (f) Average PPF at 200 ms ISI. Representative traces are shown on the right of the panel. (g) AMPA/NMDA ratio is not significantly changed by APPSw. (h) The *App* ^*s*^ mutation does not significantly change frequency of mIPSC. (i) Amplitude of mIPSC is not altered in *App* ^*s*^ rats. (j) Decay time of mIPSCs was not changed by the Swedish mutation. (k) Representative recording traces of mIPSCs. (l) Average PPF at 50 ms ISI. (m) Average PPF at 200 ms ISI. Representative traces are shown on the right of the panel. Data were analyzed by ordinary one‐way ANOVA followed by post hoc Tukey's multiple comparisons test when ANOVA showed statistically significant differences. For these experiments, we used: 6 male and 6 female *App* ^*h/h*^, *App* ^*s/h*^, and *App* ^*s/s*^ rats for glutamate recordings and 6 male and 6 female *App* ^*h/h*^, *App* ^*s/h*^, and *App* ^*s/s*^ rats for GABA recordings. The number of recordings analyzed for each group is indicated inside the bars. All data represent means ± *SEM*

The above data, together with the fact that in KI models expression of mutant genes is controlled by endogenous regulatory elements in physiological quantitative‐spatial‐temporal manner, indicate that the *App* ^*s*^ rats should be suitable to test the hypothesis that β‐processing of APP tunes up glutamate release by downregulating the intravesicular APP‐SV interactions. Thus, we studied glutamatergic synaptic transmission at the hippocampal Schaffer Collateral CA3 > CA1 synapses. First, we analyzed mEPSC. The frequency of mEPSC is in part determined by changes in release probability (P*r*) of glutamatergic SV, such that a decrease in P*r* leads to a decrease in frequency and vice versa. The frequency of mEPSC was increased in *App* ^*s/s*^ rats [ANOVA summary: *F* = 18.45, *p* < .0001 (significant = ****). Post hoc Tukey's multiple comparisons test: *App* ^*h/h*^ vs. *App* ^*s/h*^, *p* = .9634 (not significant); *App* ^*h/h*^ vs. *App* ^*s/s*^, *p* < .0001 (significant=****); *App* ^*s/h*^ vs. *App* ^*s/s*^, *p* < .0001 (significant=****)] (Figure 4a). To further test the role of β‐processing of APP in glutamate release, we examined the effect of the Swedish mutation on PPF. This form of short‐term synaptic plasticity is also determined, at least in part, by changes in P*r*, such that a decrease in P*r* leads to an increase in facilitation and vice versa. PPF was significantly decreased in both *App* ^*s/s*^ and *App* ^*s/h*^ rats [ANOVA summary of PPF at 50 ms ISI: *F* = 4.773, *p* = .0113 (significant = *). Post hoc Tukey's multiple comparisons test: *App* ^*h/h*^ vs. *App* ^*s/h*^, *p* = .0395 (significant=*); *App* ^*h/h*^ vs. *App* ^*s/s*^, *p* = .0138 (significant=*); *App* ^*s/h*^ vs. *App* ^*s/s*^, *p* = .9166 (not significant). ANOVA summary of PPF at 200 ms ISI: *F* = 0.01811, *p* = .9821 (not significant)] (Figure 4e,f), suggesting that APPSw can tune‐up glutamate release. On the whole, these data are consistent with the hypothesis that processing of APP by BACE1 increases the P*r* of glutamatergic SV.

The amplitude of mEPSCs, which is dependent on postsynaptic AMPA (α‐amino‐3‐hydroxy‐5‐methyl‐4‐isoxazole propionic acid) receptor (AMPAR‐mediated responses), was slightly, yet significantly, increased in *App* ^*s/s*^ rats [ANOVA summary: *F* = 4.586, *p* = .0183 (significant = *). Post hoc Tukey's multiple comparisons test: *App* ^*h/h*^ vs. *App* ^*s/h*^, *p *=* *.9006 (not significant); *App* ^*h/h*^ vs. *App* ^*s/s*^, *p *=* *.0253 (significant = *); *App* ^*s/h*^ vs. *App* ^*s/s*^, *p *=* *.0519 (not significant)] (Figure 4b) while the decay time was unchanged [ANOVA summary: *F* = 0.6176, *p *=* *.5460 (not significant)] (Figure 4c). To further test whether postsynaptic AMPAR‐mediated responses are increased in *App* ^*s/s*^ rats, we measured AMPAR and NMDAR‐dependent synaptic responses. Although the AMPA/NMDA ratio was slightly increased in *App* ^*s/s*^ rats, the increase did not reach statistical significance [ANOVA summary: *F* = 2.203, *p *=* *.1323 (not significant)] (Figure 4g). Thus, more in‐depth analysis is required to determine whether APPSw boosts the amplitude of AMPAR‐mediated responses.

Although GABAergic inhibitory interneurons constitute a minor fraction of hippocampal neurons (~10%), they play an important role in the regulation of the hippocampal network (Chamberland & Topolnik, 2012). Thus, we tested whether the APPSw mutation impacted GABAergic transmission. APPSw did not alter the frequency [ANOVA summary: *F* = 0.1914, *p *=* *.6433 (not significant)], amplitude [ANOVA summary: *F* = 0.4474, *p *=* *.8268 (not significant)], or decay time [ANOVA summary: *F* = 2.722, *p *=* *.0814 (not significant)] of GABAergic miniature inhibitory postsynaptic currents (mIPSC) at SC inhibitory synapses (Figure 4h–j). In addition, PPF was also not significantly affected by the *App* ^*s*^ allele [ANOVA summary of PPF at 50 ms ISI: *F* = 2.836, *p *=* *.0715 (not significant). ANOVA summary of PPF at 200 ms ISI: *F* = 2.353, *p *=* *.1088 (not significant)] (Figure 4l,m). These data are consistent with the evidence that the ISVAID and JCasp regions of APP form an interactome with glutamatergic but not GABAergic SV and that interfering with the function of ISVAID facilitates glutamate release but does not affect GABA transmission (Yao et al., 2019). Overall, the data suggest that APPSw causes an excitation/inhibition imbalance in the SC pathway, favoring excitation.

### 2.5 *App‐*Swedish rats show normal brain development and no observable neurodegeneration and AD‐like pathology at 3 months of age

**Figure 5:**
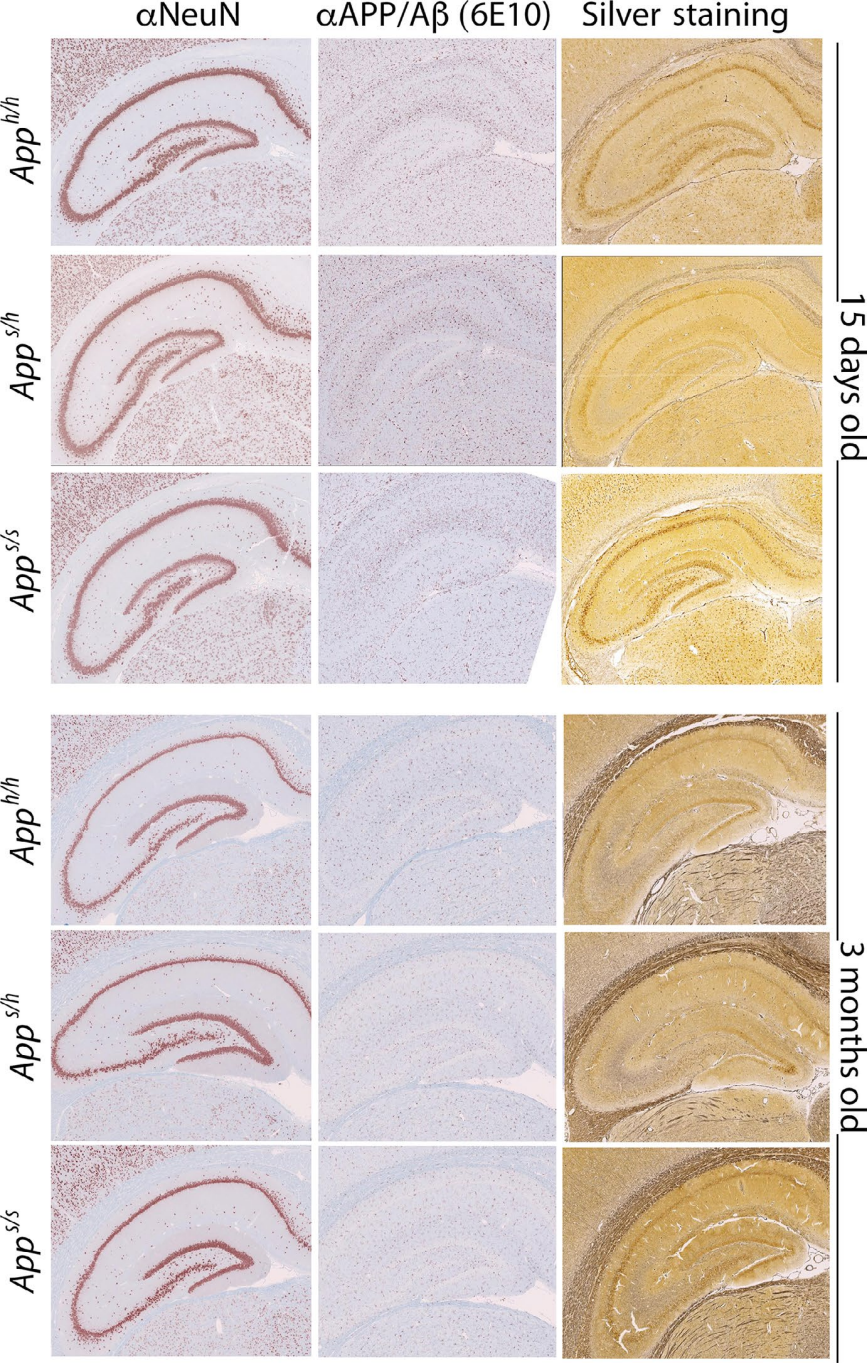
The *App* ^*s/h*^ and *App* ^*s/s*^ rats do not show AD‐like histopathology at 15 days and 3 months of age. Representative IHC and histology from the anterior hippocampus in a representative 15‐day‐ and 3‐month‐old subject for each genotype. Three female and three male rats per each genotype and age were tested

We used histological and immunohistochemical (IHC) analyses to test whether these alterations in glutamate release precede and or co‐occur with AD‐like pathology. NeuN stained tissue was used to assess neuronal density in the frontal cortex, retrosplenial, piriform, and entorhinal cortex, as well as the anterior and posterior hippocampus. A qualitative analysis showed no aberrant morphology in *App* ^*s/h*^ and *App* ^*s/s*^ rats as compared to *App* ^*h/h*^ at both ages (15 days and 3 months). The modified Bielshowski silver stain was used to identify plague structures in the tissue, along with aberrant neuronal inclusion such as tangles, and apoptotic driven cell death. No evidence of extracellular plaques, cell death, and aberrant neuronal inclusion were observed in the 3 different genotypes at both ages (Figure 5) on the same scale. Beta amyloid aggregates were further assessed using the 6E10 antibody: again, no dense plaques were evident in all animals tested (Figure 5). These results, which are not surprising given the young age of the animals analyzed, suggest that alterations in glutamatergic transmission are independent of AD‐like pathological alterations.

## 3 DISCUSSION

Human genetic data suggest that APP processing plays a significant role in the pathogenesis of both familial and sporadic dementias. Mutations in *APP* and in genes that regulate APP processing—such as *PSEN1*/*PSEN2*, γ‐secretase components, and *BRI2/ ITM2B*—cause familial dementia (De Strooper & Voet, 2012; Garringer, Murrell, D'Adamio, Ghetti & Vidal, 2010; Giliberto, Matsuda, Vidal & D'Adamio, 2009; Matsuda, Giliberto, Matsuda, McGowan & D'Adamio, 2008; Matsuda, Matsuda, Snapp & D'Adamio, 2011; Matsuda, Tamayev & D'Adamio, 2011; Matsuda et al., 2005; Tamayev, Matsuda, Arancio & D'Adamio, 2012; Tamayev, Matsuda, Fa, Arancio & D'Adamio, 2010; Tamayev, Matsuda, Giliberto, Arancio & D'Adamio, 2011; Tamayev, Giliberto et al., 2010). *BACE1* gene polymorphisms as well as increased BACE1 expression/activity are associated with sporadic dementia (Cheng et al., 2014; Hampel & Shen, 2009; Hebert et al., 2008; Holsinger, Lee, Boyd, Masters & Collins, 2006; Jo et al., 2008; Kan et al., 2005; Long, Ray & Lahiri, 2014). In contrast, humans carrying the Icelandic APP variant, which codes for an APP protein that is inefficiently cleaved by BACE1, are protected from dementia and normal cognitive decline (Jonsson et al., 2012). This genetic link between APP processing and dementia has provided some of the foundation for the “amyloid hypothesis,” which indicts Aβ as the main pathogenic factor responsible for neurodegeneration.

Yet, experimental evidence from model organisms—in conjunction with the plethora of failed clinical trials targeting Aβ production/clearance/deposition—questions the central role of amyloid peptides in dementia. Transgenic mice that express mutant APP using a tet‐Off vector systems (APPsi:tTA mice) show high amyloid burden and short/long‐term memory deficits. Suppression of APP expression after memory deficits ensue causes rapid decline in the brain levels of soluble full‐length APP, sAPPα, sAPPβ, αCTF, and βCTF and significantly improves memory deficits in spite of persisting amyloid deposits, soluble, and oligomeric assemblies of Aβ2 (Melnikova et al., 2013). BRI2‐Aβ mice produce high levels of Aβ peptides and BRI2‐Aβ1‐42 mice develop amyloid pathology that is similar to that observed in mutant human APP transgenic models (McGowan et al., 2005). Yet, BRI2‐Aβ1‐42 mice show intact cognitive performance both pre‐ and postamyloid plaque formation (Kim et al., 2013). Knock‐in models of Familial British and Danish dementia (FBD and FDD KI mice), two AD‐like dementias that are believed to be caused by other amyloidogenic moieties (ABri and ADan+Aβ42, respectively), develop long‐term potentiation (LTP) and memory deficits in the absence of amyloidosis (Giliberto et al., 2009; Tamayev, Giliberto et al., 2010; Tamayev, Matsuda et al., 2010). Memory and LTP deficits are mediated by APP and/or its non‐Aβ metabolites (Tamayev et al., 2011). Selective reduction of APP processing by BACE1 ameliorates memory and long‐term potentiation impairments (Tamayev et al., 2012); in contrast, inhibiting Aβ production worsens them (Tamayev & D'Adamio, 2012).

While the β‐processing of APP has been studied and targeted for its capacity to produce Aβ, we postulate that the β‐processing of APP may also modulate the function of the full‐length precursor protein, as well. Specifically, we have found that β‐secretase cleaves a functional domain of APP called ISVAID, which interacts with synaptic vesicle proteins. Interactomic and electrophysiology studies suggest that β‐cleavage may reduce or abolish the intravesicular interactions of APP with SV proteins, leading to facilitation of glutamate release (Yao et al., 2019). Published evidence using BACE1 inhibitors and BACE1 KO mice is consistent with this hypothesis (Filser et al., 2014; Wang et al., 2008). However, BACE1 has many substrates that function at the synapse, and off‐target effects of pharmacological and/or genetic ablation of β‐secretase activity would obscure the relative effect of the β‐processing of APP on synaptic transmission alterations. To test this hypothesis directly, we generated the *App* ^*s*^ KI rats, which carry the pathogenic Swedish *APP* mutations. APPSw, the mutant protein coded for by the Swedish *APP* allele, carries the amino acids substitutions K670N/M671L, which are localized at the NH_2_‐terminus of the β‐cleavage site of APP. Consistent with what has been observed in cell lines (Citron et al., 1992, 1994; Johnston et al., 1994), in *App* ^*s/s*^ KI rats, cleavage of APPSw by BACE1 is increased (Figure 2) without altering BACE1 activity. In parallel, these rats show augmented glutamate release at SC–CA3>CA1 pyramidal cell synapses. It is worth nothing that rats carrying one wild‐type and one Swedish allele (*App* ^*s/h*^), which genetically mimic patients since the Swedish mutation is pathogenic in heterozygosity in humans, have an intermediate phenotype. In fact, *App* ^*s/h*^ rats have only decreased PPF at 50 ms ISI, while *App* ^*s/s*^ KI rats show also increased mEPSCs frequency. These gene dosage‐dependent effects suggest that the Swedish mutation may have an accelerated pathogenic effect in humans that may carry two mutant alleles.

In contrast, the APP Swedish mutation did not significantly alter GABA release, which is consistent with the evidence that the SV‐APP‐interacting networks may be restricted to glutamatergic SV and may modulate excitatory but not inhibitory synaptic transmission (Yao et al., 2019). Thus, the Swedish APP mutation causes an excitation/inhibition imbalance, favoring excitation. In addition, our data indicate that AD‐like pathological lesions are not driving the changes in BAD‐Glu caused by the Swedish mutation.

**Figure 6:**
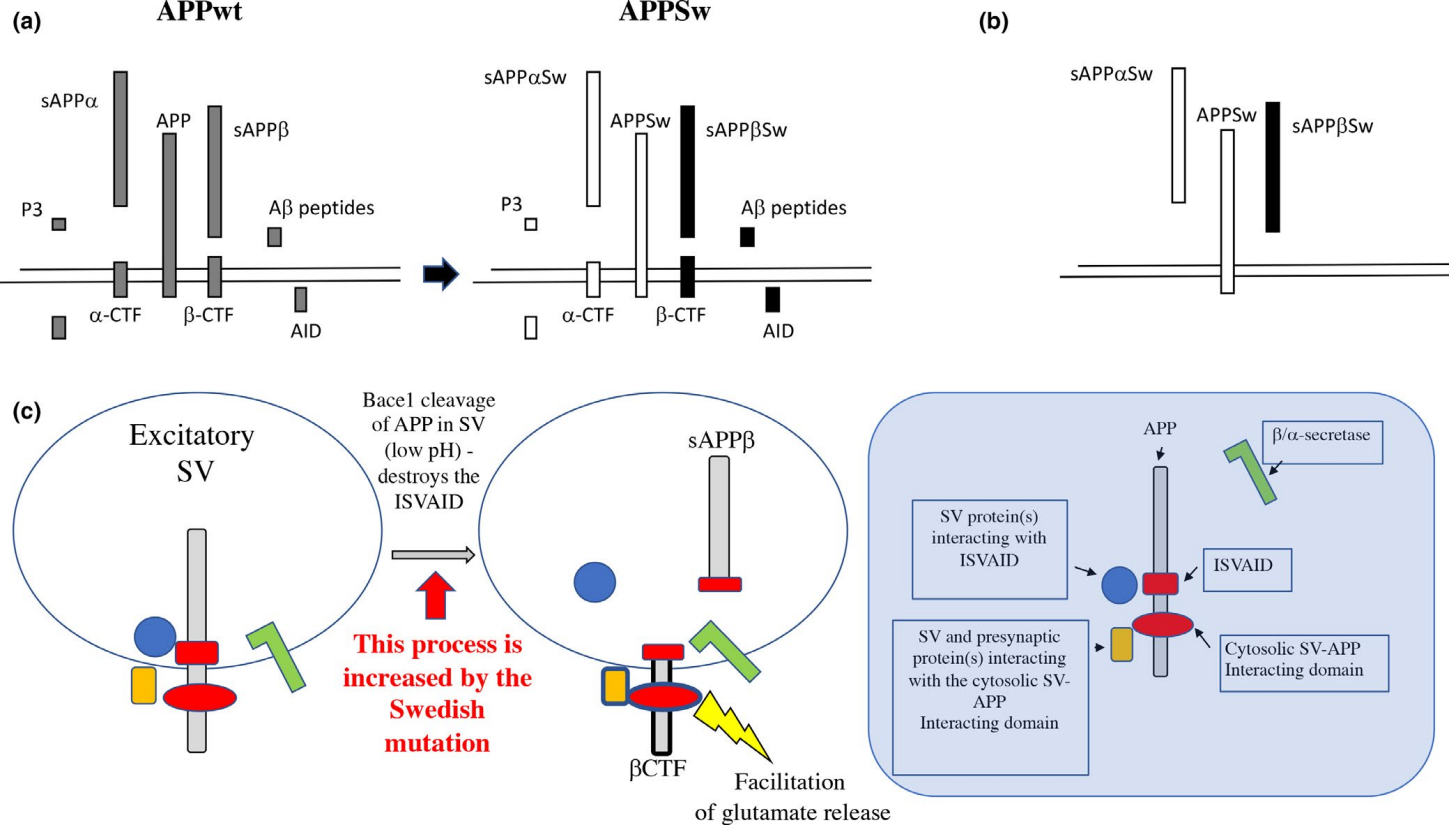
Modeling BAD‐Glu mechanisms and how the Swedish mutation may alter BAD‐Glu. (a) APP undergoes complex proteolysis. In the amyloidogenic proteolytic cascade, APP is cleaved by β‐secretase/BACE1 into sAPPβ and the COOH‐terminal fragment βCTF. Cleavage of βCTF by γ‐secretase produces Aβ peptides and the intracellular domain (AID/AICD). Alternatively, APP is processed by α‐secretase into sAPPα and the COOH‐terminal fragment αCTF. αCTF can be cleaved by γ‐secretase to produce P3 and AID/AICD (Passer et al., 2000; Sisodia & St George‐Hyslop, 2002). The Swedish mutations cause more β‐cleavage with a corresponding increase in direct and indirect metabolites (filled in black), but less α‐cleavage with a corresponding decrease in direct and indirect metabolites (filled in white). In addition, mAPP levels are reduced. The quantitative alterations in one or more of these APP metabolites can participate in BAD‐Glu dysregulation caused by the Swedish mutations. (b) The Swedish mutations cause changes in the primary sequence of several APP metabolites, including APP (APPSw), sAPPβ (sAPPβSw), and sAPPα (sAPPαSw). The qualitative alterations in one or more of these APP metabolites can participate in BAD‐Glu dysregulation caused by the Swedish mutations. (c) APP present in synaptic vesicles can interact with SV proteins and proteins regulating exocytosis via an intraluminal (ISVAID) and a cytosolic (JCasp) domain. Intraluminal and cytosolic interactions may have an opposite effect: the former tunes down glutamate release while the latter facilitates glutamate release. Cleavage of APP by BACE1 inside the ISVAID can abrogate the intravesicular interaction triggering the facilitator function of the cytosolic interaction. Increased β‐cleavage of APPSw can dysregulate BAD‐Glu dysregulation and facilitate glutamate release

What are the molecular mechanisms underlying deregulation of BAD‐Glu by APPSw? The Swedish mutation causes increased β‐processing and decreased α‐processing of APP (Figure 2). Therefore, the direct products of β‐cleavage (sAPPβ and βCTF) are increased while the metabolites formed by α‐cleavage (sAPPα and αCTF) are decreased. Since APP‐CTFs are substrates of γ‐secretase, Aβ, which is produced by γ‐cleavage of βCTF, is also increased, while P3, a metabolite produced by γ‐processing of αCTF, should be decreased—albeit we do not experimentally show this. In addition, levels of mAPP are also reduced. Thus, these metabolic alterations may contribute to dysregulation of BAD‐Glu in *App* ^*s/s*^ KI rats (Figure 6a).

Another possibility to bear in mind is that the amino acid substitutions K670N/M671L will alter the primary structure of several APP metabolites including full‐length APP, sAPPα, and sAPPβ. Although the K670N/M671L mutations do not seem to impact the intravesicular interactions of APP (Figure 3), it is possible that they may contribute to deregulation of BAD‐Glu (Figure 6b).

Finally, β‐cleavage of APP in the ISVAID may directly facilitate glutamate release via a negative modulation of intravesicular interactions, which in turn may facilitate excitatory transmission by functionally enabling the cytosolic interactions. In this model, APP would work as a fine‐tuning unit of glutamate, but not GABA, release with β‐secretase representing the rheostat (Figure 6c). Conditions that augment the rheostat activity, such as the pathogenic APP Swedish mutation, may favor excitation over inhibition, neuronal hyperexcitability, and a pro‐epileptogenic condition. Of note, unprovoked seizures occur in AD patients at rates 8‐ to 10‐fold higher than in the general population (Hauser, Morris, Heston & Anderson, 1986; Scarmeas et al., 2009) and at even higher rates in FAD cases (Cabrejo et al., 2006; Mendez & Lim, 2003; Palop & Mucke, 2016). It has also been reported that anti‐epileptic drug levetiracetam rescues cognitive deficits in MCI patients (Bakker et al., 2012). These observations are compatible with the idea that increased glutamatergic tone may have an important pathogenic role, at least in a subset of dementia patients.

All these potential mechanisms do not need to be mutually exclusive and may coincide to result in the dysregulation of BAD‐Glu seen in Swedish mutants. Future studies, including longitudinal cognitive assessment and pathology analyses of Swedish rats, are needed to assess whether this early synaptic alteration caused by APPSw underlies pathogenic mechanisms leading to neurodegeneration.

## 4 EXPERIMENTAL PROCEDURES

### 4.1 Rats and ethics statement

Rats were handled according to the Ethical Guidelines for Treatment of Laboratory Animals of the NIH. The procedures were described and approved by the Institutional Animal Care and Use Committee (IACUC).

### 4.2 Rat brain preparation

Brains were homogenized using a glass‐Teflon homogenizer (w/v = 100 mg tissue/1 ml buffer) in 250 mM sucrose, 20 mM Tris‐base pH 7.4, 1 mM EDTA, 1 mM EGTA plus protease, and phosphatase inhibitors (ThermoScientific), with all steps carried out on ice or at 4°C. Homogenates were centrifuged at 800 *g* for 10 min. Supernatant was collected and labeled S1 and used for Western analysis. Soluble fractions were generated by ultracentrifugation of S1 at 70 000 *g* for 1 hr to obtain S70 and P70. S70 was used for Western analysis of soluble APP content. Soluble fractions for ELISA were generated by solubilization of S1 with 0.1% SDS and 1% NP‐40 for 30 min rotating. Solubilized S1 was spun at 20 000 *g* for 10 m and analyzed by ELISA.

### 4.3 Generation of App KI rats

#### 4.3.1 Generation of rats carrying the App gene with the humanized Aβ sequence and rats with 7 bp deletion in App exon 16

The rat *App* gene (GenBank accession number: NM_019288.2; Ensembl: ENSRNOG00000006997) is located on rat chromosome 11. We created Long Evans rats with point mutation GGA>CGA, TTC>TAC, CGC>CAT at rat *App* locus by CRISPR/Cas‐mediated genome editing. These mutations will create a rat that carries an *App* gene coding for rat APP with the humanized Aβ sequence. The rat *App* gene comprises 18 exons, with the ATG start codon in exon 1 and TAA stop codon in exon 18; the GGA, TTC, and CGC codons are located in exon 16. Thus, exon 16 was selected as target site. gRNA targeting vector and oligo donor (with targeting sequence, flanked by 120‐bp homologous sequences combined on both sides) were designed as follows.

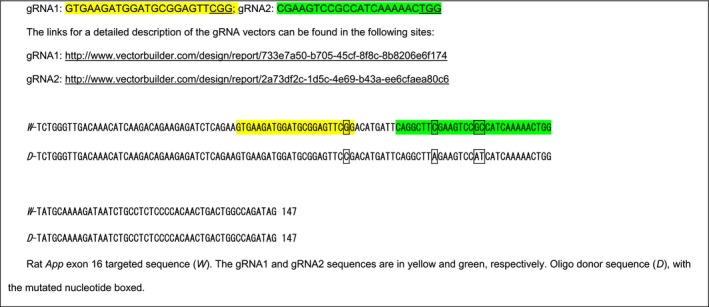

Cas9 mRNA, gRNA generated by in vitro transcription, and oligo donor were co‐injected into zygotes for production of rats carrying these knock‐in (KI) mutations by homology‐directed repair. To verify CRISPR‐induced mutation, the pups were genotyped by PCR, followed by sequence analysis. The rat *App* locus was amplified by PCR with the following specific forward (F) and reverse (R) primers: F‐CTTTCTCCAGTCTGTTTGCTTGCG; R‐GCCTGCTTCCGTGCTTCCTTT.

Cas9 mRNA, sgRNA, and oligo donor are co‐injected into zygotes, but homology‐directed repair can occur even after few cell cycles. Thus, injected rats can have a mixture of correctly targeted alleles and alleles carrying aberrant mutations or no mutations. To identify rats carrying correctly targeted *App* alleles, the PCR products were cloned into TA vectors and 10 clones were sequenced using forward primer: 5‐GTCAATGGTTTCAATCTAGGATG‐3′. This analysis showed that RatID#120 had three types of alleles:

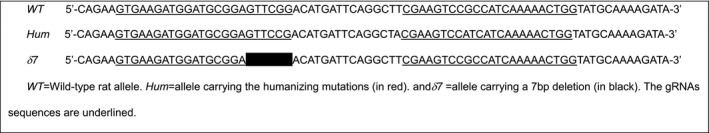

If properly spliced, the δ7 *App* allele is predicted to produce a mRNA (*App* ^δ7^ mRNA) coding for a truncated, soluble protein called APPδ7 (Figure 1a).

Thus, RatID#120 was identified as a positive chimeric founder F0‐*App* ^*h/*δ*7*^ rat.

#### 4.3.2 Off‐target analysis for gRNA1 and sRNA2

Homology‐directed repair can cause off‐target mutations in genetic sites that have high homology with the gRNAs. We identified potential off‐target sites for gRNA1 and gRNA2. Based on this analysis, RatID#120 (F0‐*App* ^*h*^ rat) has been analyzed for mutations in these most likely off‐target mutation sites. Mismatched bases are in red.

#### 4.3.3 Off‐target analysis of targeting sequence gRNA1: GTGAAGATGGATGCGGAGTTCGG

Three potential off‐target sites have been identified (mismatched bases with the targeting sequence are in red). These sites have been amplified by PCR and sequenced.

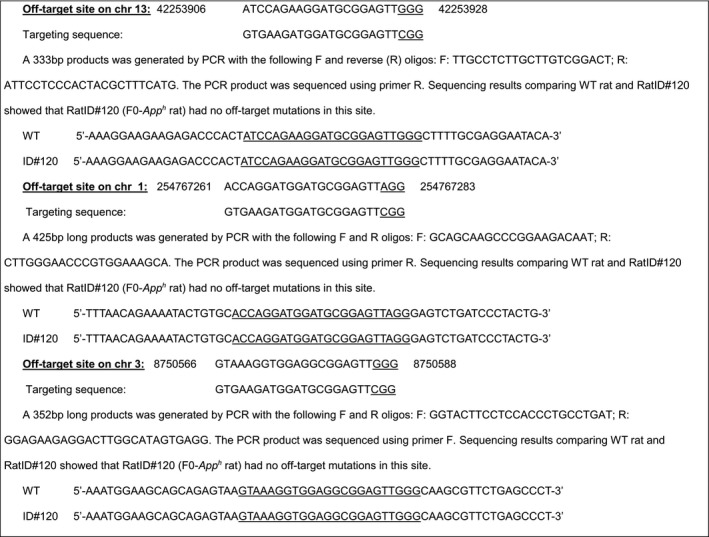

#### 4.3.4 Off‐target analysis of targeting sequence gRNA2: CGAAGTCCGCCATCAAAAACTGG

Two potential off‐target sites have been identified (mismatched bases with the targeting sequence are in red). These sites have been amplified by PCR and sequenced.

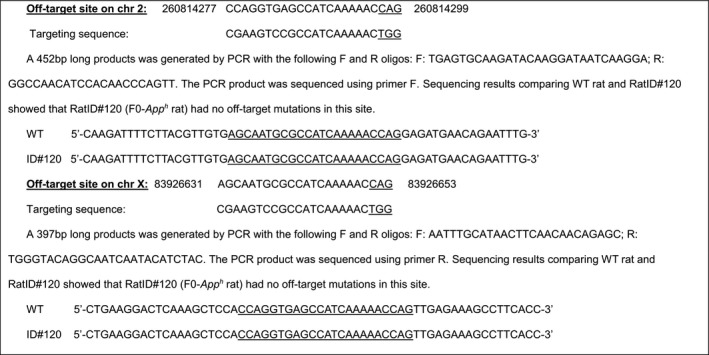

#### 4.3.5 Generation of rats carrying the App gene with the humanized Aβ sequence and the FAD Swedish mutations

We created Long Evans rats with point mutations AAG>AAT, ATG>CTG, GGA>CGA, TTC>TAC, CGC>CAT at the *App* locus by CRISPR/Cas‐mediated genome engineering**.** These mutations will create a rat that carries a humanized Aβ APP sequence plus the FAD Swedish mutation KM>NL. The AAG, ATG, GGA, TTC, and CGC codons are located in exon 16. gRNA targeting vector and oligo donor (with targeting sequence, flanked by 120‐bp homologous sequences combined on both sides) were designed as follows.

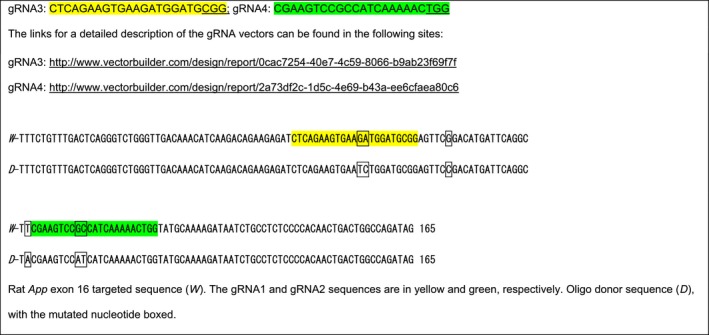

Cas9 mRNA, gRNA generated by *in vitro* transcription and oligo donor were co‐injected into zygotes for production of rats carrying these knock‐in (KI) mutations by homology‐directed repair. To verify CRISPR‐induced mutation, the pups were genotyped by PCR, followed by sequence analysis. The rat *App* locus was amplified by PCR. PCR products were cloned into TA vectors and 10 plasmids containing *App* inserts were sequenced as described above for the humanizing mutations. This analysis showed that RatID#24 had three types of alleles:

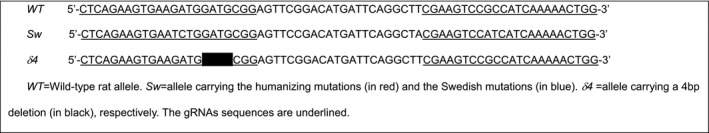

Thus, RatID#24 was identified as a positive chimeric founder (F0‐*App* ^*s*^ rat).

#### 4.3.6 Off‐target analysis of targeting sequence gRNA3: CTCAGAAGTGAAGATGGATGCGG

Three potential off‐target sites have been identified (mismatched bases with the targeting sequence are in red.) These sites have been amplified by PCR and sequenced.

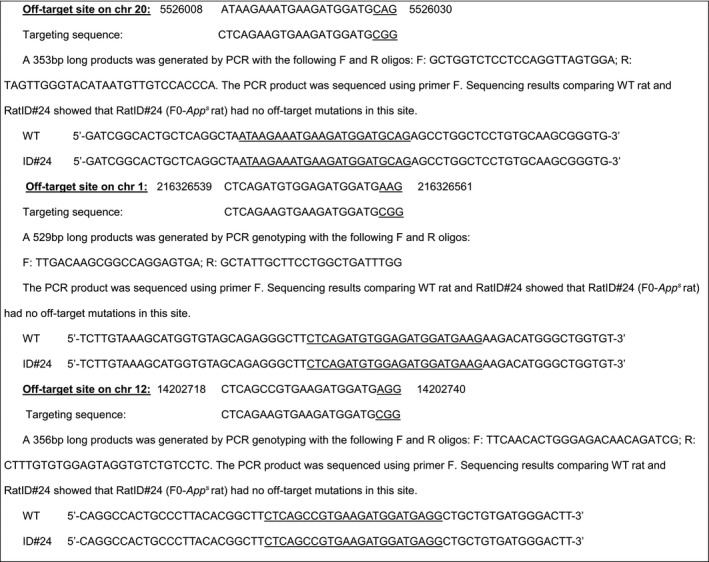

#### 4.3.7 Off‐target analysis of targeting sequence gRNA4: CGAAGTCCGCCATCAAAAACTGG

Two potential off‐target sites have been identified (mismatched bases with the targeting sequence are in red). These sites have been amplified by PCR and sequenced.

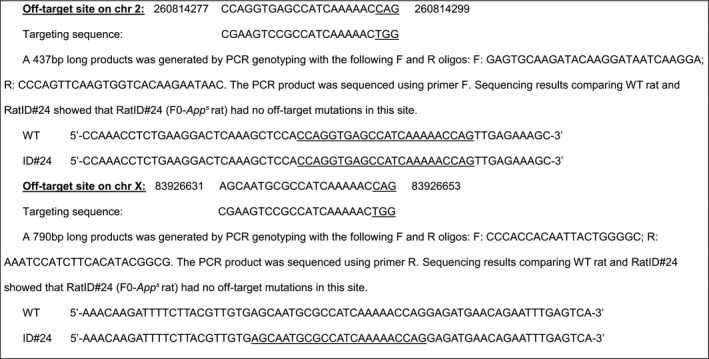

F0‐*App* ^*h/*δ*7*^ and F0‐*App* ^*s*^ rats were crossed to WT (*App* ^*w/w*^) Long Evans rats to generate F1‐*App* ^δ*7/w*^, F1‐*App* ^*h/w*^, and F1‐*App* ^*s/w*^ rats. The δ*7* mutant allele was a product of aberrant homology‐directed repair, but is useful because this mutation will either produce a truncated soluble APPδ7 protein (sAPPδ7) or a hypomorphic allele. F1‐*App* ^δ*7/w*^, F1‐*App* ^*h/w*^, and F1‐*App* ^*s/w*^ rats were crossed to WT Long Evans to generate F2‐*App* ^δ*7/w*^, F2‐*App* ^*h/w*^, and F2‐*App* ^*s/w*^ rats. These crossing were repeated three more times to obtain F5‐*App* ^δ*7/w*^, F5‐*App* ^*h/w*^, and F5‐*App* ^*s/w*^ rats. The probability that F5 rats carry unidentified off‐target mutations (except those, if present, on Chr. 11) is ~1.5625%. Male and female F5‐*App* ^δ*7/w*^, F5‐*App* ^*h/w*^, and F5‐*App* ^*s/w*^ rats were crossed to obtain *App* ^δ*7/*δ*7*^ *, App* ^*h/h*^, and *App* ^*s/s*^ rats.

### 4.4 Brain slice preparation

Rats were deeply anesthetized with isoflurane and intracardially perfused with an ice‐cold cutting solution containing (in mM) 120 choline chloride, 2.6 KCl, 26 NaH CO3, 1.25 NaH2PO4, 0.5 CaCl2, 7 MgCl2, 1.3 ascorbic acid, 15 glucose, prebubbled with 95% O2/5% CO2 for 15 min. The brains were rapidly removed from the skull. Coronal brain slices containing the hippocampal formation (350 μm thick) were prepared in the ice‐cold cutting solution bubbled with 95% O2/5% CO2 using Vibratome VT1200S (Leica Microsystems) and then incubated in an interface chamber in ACSF containing (in mM): 126 NaCl, 3 KCl, 1.2 NaH2PO4; 1.3 MgCl2, 2.4 CaCl2, 26 NaHCO3, and 10 glucose (at pH 7.3), bubbled with 95% O2 and 5% CO2 at 30°C for 1 hr and then kept at room temperature. The hemi‐slices were transferred to a recording chamber perfused with ACSF at a flow rate of ~2 ml/min using a peristaltic pump. Experiments were performed at 28.0 ± 0.1°C.

### 4.5 Whole‐cell electrophysiological recording

Whole‐cell recordings in the voltage‐clamp mode(‐70 mv) were made with patch pipettes containing (in mM): 132.5 Cs‐gluconate, 17.5 CsCl, 2 MgCl2, 0.5 EGTA, 10 HEPES, 4 ATP, and 5 QX‐314, with pH adjusted to 7.3 by CsOH. Basal synaptic responses were evoked at 0.05 Hz by electrical stimulation of the Schaffer collateral afferents using concentric bipolar electrodes. Excitatory postsynaptic currents (EPSCs) were recorded in ACSF containing 15 μM bicuculline methiodide to block GABA‐A receptors. For recording of paired‐pulse ratio (PPR), paired‐pulse stimuli with 50 or 200 ms interpulse interval were given. The PPR was calculated as the ratio of the second EPSC amplitude to the first. For recording of AMPA/NMDA ratio, the membrane potential was held at −70 mV to record only AMPAR current, and then, the membrane potential was turned to +40 mV to record NMDAR current. Mini EPSCs were recorded by maintaining neurons at −70 mV with ACSF containing 1 μM TTX and 15 μM bicuculline methiodide to block action potentials and GABA‐A receptors, respectively, and analyzed using mini Analysis Program.

Mini IPSCs were recorded with patch pipettes containing (in mM): 135 KCl, 2 MgCl2, 0.1 EGTA, 10 HEPES, 2 Na2ATP, 0.2 Na2GTP (PH 7.3, osmolarity 290–310 mOsm) with 1 μM TTX and AMPA receptor antagonist NBQX (10 μM, Tocris) in perfusing ACSF. Data were collected and analyzed using the Axopatch 700B amplifiers and pCLAMP10 software (Molecular Devices).

### 4.6 Western analysis

Protein content quantified by Bradford analysis. 15 μg of protein from each fraction was brought to 15 μl with PBS and LDS sample buffer‐10% β‐mercaptoethanol (Invitrogen NP0007) to 1×, boiled for 1 m, cooled on ice, and loaded on a 4%–12% Bis‐Tris polyacrylamide gel (Bio‐Rad 3450125). Proteins were transferred onto nitrocellulose at 25 V for 7 min using the Trans‐blot Turbo system (Bio‐Rad) and visualized by red Ponceau staining. Membranes were blocked 30 min in 5%‐milk (Bio‐Rad 1706404) and washed extensively in PBS/Tween‐20‐0.05%, and primary antibody was applied overnight at 4°C at 1:1000 dilution in blocking solution (Thermo 37573). The following antibodies were used: Y188 (APP‐C terminus, Abcam ab32136), 6E10 (APP‐Aβ_1‐16_, BioLegend 803001), sAPPα (sAPPα‐C terminus, IBL 2B3), M3.2 (APP‐Aβ_1‐16_, Biolegend 11465), sAPPβ (sAPPβ‐C terminus, Antibodies Online ABIN927102 and Covance Catalog# SIG‐39138),), sAPPβ‐Sw (sAPPβ‐Sw‐C terminus, IBL 6A1), s22C11 (APP N terminus, EMD‐Millipore Mab348), Synaptotagmin1B (Synaptotagmin1B a.a. 171‐187, Synaptic Systems 110402), Synaptophysin (Synaptophysin a.a. ~230, Cell Signaling 5461), Vamp2 (Vamp2 a.a. 2‐17, Synaptic Systems 104202), and Vglut1 (Vglut1 a.a. 456‐560, Synaptic Systems 135 303). Anti‐mouse (Southern Biotech, OB103105) and a 1:1 mix of anti‐rabbit (Southern Biotech, OB405005) and anti‐rabbit (Cell Signaling, 7074) were diluted 1:1000 in 5%‐milk and used against mouse and rabbit primary antibodies for 30 min, RT, with shaking. Blots were developed with West Dura ECL reagent (Thermo, PI34076) and visualized on a ChemiDoc MP Imaging System (Bio‐Rad). Signal intensity was quantified with Image Lab software (Bio‐Rad). Data were analyzed using Prism software and represented as mean ± *SEM*.

### 4.7 Immunohistochemistry (IHC) and Silver staining

#### 4.7.1 Staining tissue preparation and sectioning

Rat brain tissue was fixed by intracardiac perfusion with PBS followed by PFA and stored in 70% ethanol. Olfactory bulb and cerebellum tissue were dissected and processed in a single cassette; the cerebrum was trisected into three equivalent slabs and laid out to expose three coronal cross sections. All tissues were dehydrated through graded ethanol and xylene, infiltrated with paraffin wax, and embedded in paraffin block. Each block was sectioned into 15 cross sections targeting the frontal cortex at the level of the isthmus of the corpus callosum (CC), anterior and posterior regions of the hippocampus.

#### 4.7.2 Immunohistochemistry (IHC) staining

IHC staining was performed in accordance with Biospective Standard Operating Procedure (SOP) # BSP‐L‐06. Slides were manually de‐paraffinized and rehydrated prior to the automated immunohistochemistry. Slides initially underwent antigen retrieval, either heat‐induced epitope‐retrieval (HIER), or formic acid treatment. HIER was performed by incubation in citrate buffer (pH 6.0) and heating to 120°C under high pressure for a period of 10 min. Formic acid treatment was 15‐min incubation in 80% formic acid, followed by washing in water and TBS‐T. All IHC studies were performed at room temperature on a Lab Vision Autostainer using the REVEAL Polyvalent HRP‐AEC detection system (Spring Bioscience). Antigen retrieval was performed as outlined in Table 1, followed by immunohistochemical staining. Briefly, slides were incubated sequentially with hydrogen peroxide for 5 minutes, to quench endogenous peroxidase, followed by 5 minutes in Protein Block, and then incubated with primary, antibodies as outlined in Table 1. Antibody binding was amplified using the Complement reagent (20 min), followed by a HRP‐conjugate (20 min), and visualized using the AEC chromogen (20 minutes). All IHC sections were counterstained with Acid Blue 129 and mounted with aqueous mounting medium (Zehntner, Chakravarty, Bolovan, Chan & Bedell, 2008).

**Table 1:** List of abbreviations. The numbering of APP refers to the isoform of 695, which is the main CNS form for APP

ISVAID	Intraluminal SV‐APP Interacting Domain of APP
JCasp	Intracytosolic APP domain that terminates (C terminus) at Asp664, where caspases cleave APP (caspases cleave APP between Asp664 and Ala665)
Ex/TM	Intraluminal/extracellular APP fragment that with N terminus at Glu589 and C terminus at Gly622. This sequence contains the ISVAID domain
βCTF	Membrane‐bound C‐terminal fragment of APP produced by β‐secretase
αCTF	Membrane‐bound C‐terminal fragment of APP produced by α‐secretase
sAPPβ	Soluble N‐terminal fragment of APP produced by β‐secretase. Soluble N‐terminal fragment from APPh is sAPPβh. Soluble N‐terminal fragment from APPsw is sAPPβsw
sAPPα	Soluble N‐terminal fragment of APP produced by α‐secretase. Soluble N‐terminal fragment from APPsw is sAPPαsw

**Table:** 

Target	Antibody	Antigen Retrieval	Dilution
Neurons	NeuN, Mouse monoclonal A60, Millipore	Citrate HIER	1:200
APP/Aβ	1‐16 Aβ, Mouse monoclonal 6E10, Biolegend	80% Formic Acid	1:100

#### 4.7.3 Modified Bielshowski silver staining

The slides were manually de‐paraffinized and rehydrated prior to histological staining. Rehydrated tissue was immersed in preheated silver nitrate solution (40°C) for 15 min, followed by a deionized water rinse and an incubation in ammoniacal sliver solution at 40°C for 10 min (American Master Tech). Silver deposition was performed in the developer solution for a period of 15 min, and once a golden brown tissue stain was achieved, the development was stopped by sequential incubations in ammonium water then 5% sodium thiosulfate (American Master Tech). The stained tissue sections were dehydrated in xylene and mounted in Permount (VWR) and cover‐slipped.

#### 4.7.4 Image analysis of IHC sections

The IHC and histology slides were digitized using an Axio Scan.Z1 digital whole‐slide scanner (Carl Zeiss). The images underwent quality control (QC) review and final images transferred to the Biospective server for qualitative image analysis. All qualitative assessments were performed blinded to the tissue genotype.

### 4.8 Elisa

Aβ_40_ and Aβ_42_ content of 0.1% SDS/1% NP‐40‐solubilized S1 homogenates from brains taken from 21‐day‐old rats were measured, respectively, with human β amyloid (1‐42) ELISA Kit – High Sensitive (Wako) and Human β Amyloid (1‐40) ELISA Kit II (Wako), according to the manufacturer's instructions. Absorbances at 450 nm were read on an xMark Spectrophotometer (Bio‐Rad). Data were analyzed using Prism software and represented as mean ± *SEM*.

### 4.9 Rt–pcr

Total brain RNA was extracted from P21 rat pups with RNeasy RNA Isolation kit (Qiagen 74104) and used to generate cDNA with a High‐Capacity cDNA Reverse Transcription Kit (Thermo 4368814). 50 ng cDNA, TaqMan™ Fast Advanced Master Mix (Thermo 4444556), and the appropriate TaqMan (Thermo) probes were used in the real‐time polymerase chain reaction. Samples were analyzed on a Roche LightCycler 2.0 Thermal Cycler, and relative RNA amounts were quantified using LinRegPCR software (hartfaalcentrum.nl). The probe Rn00570673_m1 (exon junctions 11‐12, 12‐13, and 13‐14) was used to detect rat App, and samples were normalized to Gapdh levels, as detected with Rn01775763_g1 (exon junctions 2‐3, and 7‐8).

### 4.10 Experimental design and statistical analysis

Levels of *App* mRNA (Figure 1b) were measured in 21‐day‐old *App* ^*w/w*^, *App* ^δ*7/*δ*7*^ *, App* ^*h/h*^, and *App* ^*s/s*^ rats (two females and three males for each genotype). The Western blots shown in Figure 1e–g were performed using brain lysates obtained from 1 male and 1 female 21‐day‐old *App* ^*w/w*^, *App* ^δ*7/*δ*7*^ *, App* ^*h/h*^, and *App* ^*s/s*^ rats. The Western blots shown in Figure 1h–k were performed using soluble brain fractions from one 21‐day‐old male *App* ^δ*7/*δ*7*^ *, App* ^*h/h*^, and *App* ^*s/s*^ rat. The Western blots shown in Figure 2 were performed using protein extracts obtained from 21‐day‐old *App* ^*h/h*^ and *App* ^*s/s*^ rats (2 females and 3 males for each genotype). The slices used for electrophysiology studies were obtained from 6‐ to 8‐week‐old rats. For the experiments shown in Figure 3, we used: (a) 4 male and 4 female *App* ^*h/h*^ rats for the control group C; (b) 4 female and 4 male *App* ^*h/h*^ rats for the Ex/TM‐Sw group; (c) 5 female and 5 male *App* ^*h/h*^ rats for the Ex/TM group. The recordings shown in Figure 5a, b, c, d, f, and g were obtained from: 6 male and 6 female rats for each genotype (*App* ^*h/h*^, *App* ^*s/h*^ and *App* ^*s/s*^). The recordings shown in Figure 4h, i, k, l, and m were obtained from: (a) 6 male and 6 female *App* ^*h/h*^ rats; (b) 6 male and 7 female *App* ^*s/h*^ rats; (c) 5 male and 4 female *App* ^*s/s*^ rats. For the Histology/IHC studies shown in Figure 5, we used 3 rats per age per genotype (2 female and one male).

Statistical significance was evaluated using (a) ordinary one‐way ANOVA followed by Post hoc Tukey's multiple comparisons test when applicable (i.e. when the ordinary one‐way ANOVA test showed statistical significance) for experiments shown in Figures 1, 3, and 4; (b) Student's *t* test for experiments shown in Figure 2. Statistical analysis was performed with GraphPad Prism v8 for Mac. Significant differences were accepted at *p *<* *.05.

## AUTHOR CONTRIBUTIONS

M.D.T., W.Y., and L.D. designed the experiments. W.Y. performed the electrophysiology experiments. L.D. designed the KI rats. M.D.T. and L.D. analyzed and characterized the KI rats. L.D. wrote the manuscript. All authors provided editorial comments.

## CONFLICT OF INTEREST

LD and WY are inventors on U.S. Provisional Patent Application Number: 62/831,287 which includes ISVAID.

5

## Data Availability

The data that support the findings of this study are available from the corresponding author upon reasonable request.
